# ﻿A new species of *Neotropiconyttus* Kirkaldy (Hemiptera, Reduviidae) in a cacao plantation from the Colombian Napo Province, including a key to species, and taxonomic notes of the genus

**DOI:** 10.3897/zookeys.1207.120663

**Published:** 2024-07-19

**Authors:** Jean Gamboa, Hélcio R. Gil-Santana, Armando Gamboa-Tabares, Eidy Martínez-Viuche, Francisco Serna

**Affiliations:** 1 Laboratorio de Entomología Universidad de la Amazonia LEUA, Facultad de Ingeniería, Universidad de la Amazonia, sede Centro, Carrera 11, 5-69, Florencia, Caquetá, Colombia Universidad Nacional de Colombia Bogotá Colombia; 2 Museo Entomológico UNAB, Facultad de Ciencias Agrarias, Universidad Nacional de Colombia, sede Bogotá, Carrera 30, 45-03, Bogotá D.C., Colombia Universidad de la Amazonia Florencia Colombia; 3 Laboratório de Diptera, Instituto Oswaldo Cruz, Av. Brasil, 4365, 21040-360, Rio de Janeiro, Brazil Laboratório de Diptera, Instituto Oswaldo Cruz Rio de Janeiro Brazil

**Keywords:** Assassin bugs, *Graptocleptes*, Heteroptera, *Hiranetis*, identification, Neotropical

## Abstract

A new species of the genus *Neotropiconyttus* Kirkaldy, 1909 (Hemiptera: Heteroptera: Reduviidae: Harpactorinae: Harpactorini) is described and illustrated. *Neotropiconyttusarmandoi* Gamboa & Gil-Santana, **sp. nov.** represents the first record of the genus for the Province of Napo in Colombia, and the first description of a male individual in the genus. The male specimen representing the new species was collected on a leaf of cacao (*Theobromacacao* L.-Malvaceae). Its remarkable similarity in external coloration and structure with that of the true bug *Monaloniondissimulatum* Distant, 1883 (Hemiptera: Miridae) inhabiting cacao agroforestry systems suggests that the new species could be part of a mimetic complex that incorporates phytophagous and predator bugs. Comments and figures of type specimens of *Neotropiconyttusalboannulatus* (Stål, 1855) and *Neotropiconyttusdama* (Burmeister, 1838), and a key to the species of the genus are also provided.

## ﻿Introduction

In the Neotropics, the tribe Harpactorini of the subfamily Harpactorinae is the most diverse group within Reduviidae, with approximately 53 genera in the Neotropics (Forero 2011; Gil-Santana 2015; Gil-Santana et al. 2015, 2017; Gil-Santana and Oliveira 2023). Different species of bugs (Hemiptera: Harpactorini), with bees and wasps, have been recognized as being involved in mimicry systems regarding the general body form, wing coloration, and characteristics concerning physical proportions (e.g., Champion 1899; Elkins 1969; Maldonado and Lozada 1992; Hogue 1993; Gil-Santana 2008, 2015, 2016, 2022; Gil-Santana et al. 2013, 2015, 2017; Castro-Huertas and Forero 2021). Maldonado and Lozada (1992) presented a key to Neotropical wasp-mimicking Harpactorinae genera, which, in their view, helps to quickly sort out specimens from unidentified material, although this is a somewhat artificial way of grouping genera. The most updated version of their key was published by Gil-Santana and Oliveira (2023), where they included the following genera: *Acanthischium* Amyot & Serville, 1843, *Coilopus* Elkins, 1969, *Graptocleptes* Stål, 1866, *Hiranetis* Spinola, 1837, *Myocoris* Burmeister, 1835, *Neotropiconyttus* Kirkaldy, 1909, *Parahiranetis* Gil-Santana, 2015, *Quasigraptocleptes* Gil-Santana & Oliveira, 2023, and *Xystonyttus* Kirkaldy, 1909. Gil-Santana and Oliveira (2023) also summarized the literature regarding these genera.

Maldonado and Lozada (1992) recognized *Neotropiconyttus* (Hemiptera, Heteroptera, Reduviidae, Harpactorinae, Harpactorini) among these genera when they described *N.heminigra* Maldonado & Lozada, 1992 and presented a table with a set of color characters to differentiate the species of *Neotropiconyttus*. However, the herein second author (HRG-S) examined the type specimens of *N.dama* (Burmeister, 1838) currently deposited at the Museum für Naturkunde Berlin, Leibniz Institute for Evolution and Biodiversity Science, Berlin, Germany (**MFNB**), and concluded that there were some errors in Maldonado and Lozada´s table, compromising the recognition of *N.dama*. For example, they recorded the pronotum and mesosternum as blackish instead of reddish. Therefore, in this study, a key based on the table published by Maldonado and Lozada (1992) was elaborated on the species of the genus, allowing a clearer-cut recognition of them.

On the other side, Kirkaldy (1909) created *Neotropiconyttus* as a new name for “*Amaurosphodrus* Stål, 1872” with [*N.*] *alboannulatus* as the type species of the genus, even though *Myocorisdama* had been described earlier (Burmeister, 1838). It is uncertain who first established the combination *Neotropiconyttusdama*, but the earliest reference to this combination is found in the catalog of Wygodzinsky (1949).

Descriptions of *N.dama* and *N.alboannulatus* were based on color features alone, and those of *N.heminigra* on color, shape, and measurements of various anatomical structures (Burmeister 1838; Stål 1855; Maldonado and Lozada 1992). Both male and female genitalia have remained undescribed for all species.

General appearance and coloration make the species of assassin bugs belonging to *Neotropiconyttus* mimetic to the true bugs *Monalonion* spp. (Hemiptera: Miridae), as well as with wasps belonging to Ichneumonidae and Braconidae (Hymenoptera). Moreover, some species of *Graptocleptes* and *Neotropiconyttus* are highly similar in shape and color. As to the living habitats of *Neotropiconyttus*, no details are included in their species descriptions.

Seeking to recognize the natural insect enemies of *Monalonion* spp. and their corresponding natural biology in cacao (*Theobromacacao* L., Malvaceae) plantations in Southeastern Colombia, the research project “Study of diversity, population dynamic and biotic potential of predators and parasitoids controlling true bugs of the genus *Monalonion* Herrich-Schäffer, 1850 in cacao plantations in the states of Huila and Caquetá, Florencia” was carried out. In this work, the first record of the genus *Neotropiconyttus* for the Napo province in Colombia and the description of a new species of the genus are included.

Besides describing *Neotropiconyttusarmandoi* sp. nov. and improving the knowledge of the species of *Neotropiconyttus*, photographs deploying the diverse coloration of the syntypes of *N.alboannulatus* and *N.dama*, are provided. The female holotype of *N.heminigra* was not located in any collection. All information about the species is considered, following the original description by Maldonado and Lozada (1992).

## ﻿Materials and methods

In 251 farms with cacao plantations in the states of Huila and Caquetá, Colombia, 5,401 Reduviidae specimens were collected, of which only one specimen matched the description of the genus *Neotropiconyttus*. The single specimen was collected on a leaf of a *T.cacao* tree. The individual was collected employing an entomological net, then placed into a 30 ml plastic bottle containing ethyl alcohol 96% and transported to Laboratorio de Entomología Universidad de la Amazonia (LEUA) in Florencia (Caquetá, Colombia). Curatorship of the specimen, which was point-mounted, was carried out following the protocols established in the LEUA insect collection.

**Figures 1–4.**
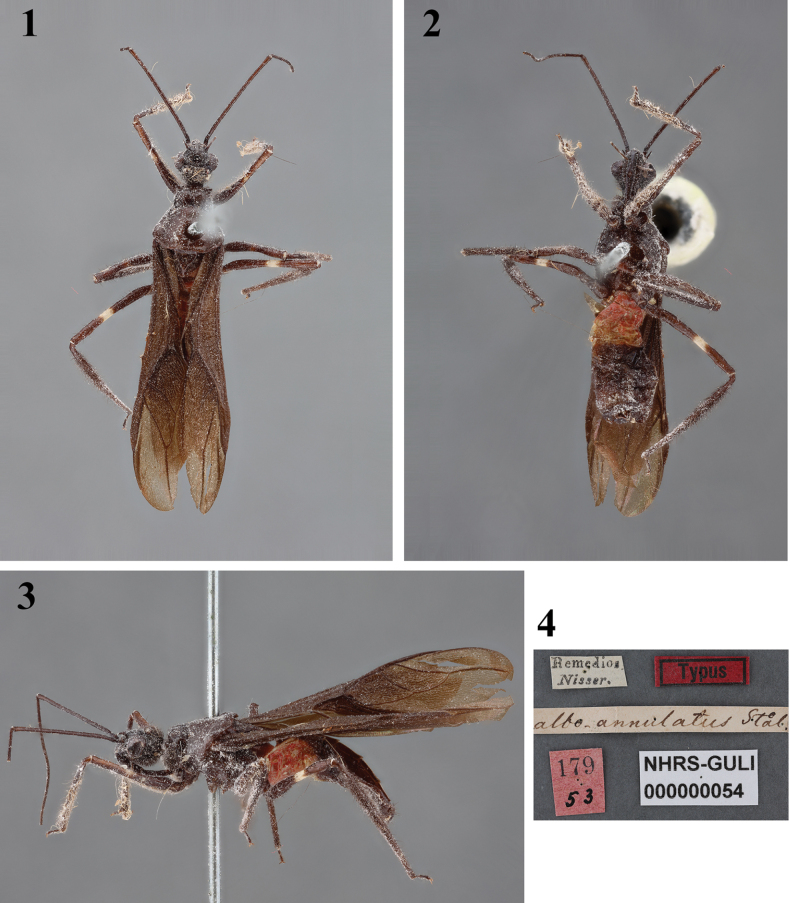
*Neotropiconyttusalboannulatus* (Stål, 1855), syntype, female, deposited in NHRS, catalog number NHRS-GULI000000054, photographs provided by Gunvi Lindberg, 2023 of Naturhistoriska Riksmuseet [Swedish Museum of Natural History] and made available by this institution under the Creative Commons Attribution 4.0 International Public License, CC-BY 4.0, https://creativecommons.org/licenses/by/4.0/legalcode**1** dorsal view **2** ventral view **3** lateral view **4** labels.

Images of a female syntype of *Neotropiconyttusalboannulatus* (Stål, 1855) (Figs 1–4), deposited in the Swedish Museum of Natural History, Stockholm, Sweden (**NHRS**) were provided by Gunvi Lindberg with the copyright belonging to the NHRS. Three female syntypes of *Neotropiconyttusdama* (Burmeister, 1838), deposited in the Hemimetabola Collection of the MFNB, were directly examined and photographed by HRG-S (43‒51) in 2015. The images were taken utilizing a Nikon D5200 digital camera with a Nikon Macro lens of 105 mm.

The identification of the specimen into the genus *Neotropiconyttus* was carried out employing the taxonomic keys proposed by Maldonado and Lozada (1992), Gil-Santana (2015), and Gil-Santana and Oliveira (2023). Additionally, comparisons of the specimen found in the current study with photographs of the female syntype of *N.alboannulatus* (Figs 1–4), available on the website of the NHRS (http://www2.nrm.se/en/het_nrm/a/neotropiconyttus_alboannulatus.html), and examination of female syntypes of *N.dama* deposited in the MFNB (Figs 43–51) by the second author (HRG-S) allowed confirm this finding.

Images and measurements of the type-specimen of *N.armandoi* sp. nov. described in this work were taken using a LEICA M205A stereomicroscope (Figs 5–16, 40–42, 56) and a HITACHI TM4000Plus II environmental scanning electron microscope (Figs 17–32). A distribution map of the species of the genus *Neotropiconyttus* was designed using of the software QGIS v. 3.26.2 (Fig. 52). Pygophore and aedeagus were drawn using the software CorelDRAW v. 2022 (Figs 33–39). All figures were prepared utilizing the software Photoshop 2023 v. 24.0.

For the morphological description, an Olympus SZ51 stereomicroscope was utilized. The pygophore was extracted employing forceps and pins and placed into a NaOH 20% solution for 24 hours. Dissected structures were then studied immersed in glycerol. After all dissections and imaging of the male genitalia portions, they were placed into a microvial attached to the bottom of the specimen pin. General terminology follows Schuh and Weirauch (2020). Male genitalia terminology follows Gil-Santana et al. (2013, 2017), Gil-Santana (2016), and Gil-Santana and Oliveira (2023). The holotype was housed in the LEUA Collection.

When describing label data, a slash (/) separates the lines, and a double slash (//) separates different labels. Comments or translations of label data into English are provided in square brackets ([]).

## ﻿Results

### ﻿Taxonomy

**Subfamily Harpactorinae**

**Tribe Harpactorini**

#### ﻿Genus Neotropiconyttus

Taxon classificationAnimaliaHemipteraReduviidae

Kirkaldy, 1909

D9A758E6-B1D7-5CBE-82C7-F4147517FAE3

Neotropiconyttus Kirkaldy, 1909: 388 [as a new name for Amaurosphodrus Stål, 1872]; Wygodzinsky 1949: 42 [catalog]; Putshkov and Putshkov 1985: 53 [catalog]; Maldonado 1990: 241 [catalog]; Maldonado and Lozada 1992: 162 [comments on diagnostic characteristics], 165 [in key]; Gil-Santana 2015: 30 [citation as a wasp-mimetic genus], 37 [in key]; Gil-Santana 2016: 92 [citation as a wasp-mimetic genus]; Gil-Santana et al. 2017: 41 [citation as a wasp-mimetic genus]; Gil-Santana and Oliveira 2023: 164 [citation as a wasp-mimetic genus], 200 [in key].

##### Type species.

[*Zelus*] *alboannulatus* Stål, 1855 by original designation, Kirkaldy, 1909: 388.

##### Diagnosis.

*Neotropiconyttus* may be separated from other wasp-mimicking genera by the following set of characters: Head fairly setose to very densely setose, especially on ventral and postocular portions, postantennal spines curved and directed forward; pronotum not inflated; scutellum visible from above; fore femora thicker only basally; fore tibiae straight.

##### Description.

Integument smooth. ***Head***: gibbous, large, approximately as long as wide across eyes (neck excluded); with sparse long and short, straight or somewhat curved blackish setae; the latter much denser, forming pubescence of long blackish thick setae on postocular portion and gula. Clypeus straight in dorsal view, curved in lateral view. Antennal insertion at level of upper 1/3 of eye; scape straight, shiny; pedicel straight or somewhat curved; basiflagellomere slightly curved or straight; in [known] males somewhat thickened in basal ~ 1/2; distiflagellomere slightly curved and thinner than the other segments. Postantennal spines strongly curved and directed forward. Eyes globose, glabrous, projecting laterally, prominent in dorsal view, close to dorsal margin of head; reaching or not reaching ventral margin of head. Interocular sulcus thin and shallow, curved laterally. Just anterior to it, on midline, a small oval fossa followed anteriorly by a very short thin shallow median sulcus, which sometimes is not evident. Ocelli and portion between them elevated, the former somewhat closer to eyes than to each other. Labium stout, curved, reaching prosternum approximately at proximal part of its distal 1/3; segment II (first visible) thickest and longest, straight, reaching level of distal 1/3 of eye or its posterior margin; segment IV shortest, triangular, tapering. Neck thin. ***Thorax***: Anterior collar narrow; anterolateral angles prominent, subtriangular. Transverse sulcus not deep, interrupted before middle by a pair of submedian shallow carinae; slightly curved laterally. Mid-longitudinal sulcus on fore lobe of pronotum moderately deep; disc of hind lobe smooth; lateral longitudinal sulci well marked at posterior 1/2 to posterior 2/3 of hind lobe of pronotum. Humeral angle slightly or not elevated, rounded at lateral margin. Scutellum with margins elevated, apex rounded. Legs: coxae globose; femora and tibiae slender, elongated, and generally straight. Fore femur shorter than head and pronotum together, thickened at basal portion and somewhat curved at midportion; middle and hind femora slightly thickened basally, sometimes dilated subapically and slightly narrower approximately at median portion where distal pale annuli may be located; apices of all femora with a pair of lateral small tubercles. Fore tibiae thickened apically, where there is a dorsal spur and a mesal comb. Hemelytra long, surpassing abdomen by ~ 1/2 length of membrane. ***Abdomen***: elongated; spiracles rounded.

#### ﻿ Neotropiconyttus alboannulatus

Taxon classificationAnimaliaHemipteraReduviidae

(Stål, 1855)

3C333E1A-72D3-53A6-A744-244FE1246BB1

Figs 1–4

Zelus alboannulatus Stål, 1855: 189 [description].
Amaurosphodrus albo-annulatus ; Stål 1867: 297 [description], 1872: 82 [catalog]; Lethierry and Severin 1896: 178 [catalog]; Champion 1899: 283 [citation, comments], tab. XVII, figs 15, 15a.
Zelus albo-annulatus ; Walker 1873: 136 [catalog].
Neotropiconyttus alboannulatus ; Kirkaldy 1909: 388 [as the type of Amaurosphodrus Stål, 1872 in the new combination]; Wygodzinsky 1949: 42 [catalog]; Maldonado 1990: 241 [catalog]; Maldonado and Lozada 1992: 165 [comparison with other species of the genus based on color characteristics]; Froeschner 1999: 207 [catalog].

##### Distribution.

Colombia.

##### Notes.

*Neotropiconyttusalboannulatus* was described based on (a) female specimen (s) from Colombia (Stål 1855) (Figs 1–3). The female “Typus” currently deposited in NHRS is considered a syntype, according to Art. 73.2 of the ICZN (1999). This specimen has a label with the locality “Remedios”, written by Stål (1855) as the original information. Therefore, according to Art. 76.1 of the ICZN (1999), “Remedios” is considered the type locality of *N.alboannulatus*. Further, the species was also recorded in Mexico (Walker 1873) and Panama (Champion 1899). The latter author observed only females similar to the type specimen.

##### Morphological remarks.

Length ~ 9.5 mm. The so far recorded specimens are generally blackish with small whitish annuli on median portion of femora, somewhat larger on hind legs, and the anterior ~ 1/2 of the ventral surface of the abdomen reddish.

#### ﻿ Neotropiconyttus armandoi

Taxon classificationAnimaliaHemipteraReduviidae

Gamboa & Gil-Santana sp. nov.

3C800A94-8332-5AE4-9780-6335AA363037

https://zoobank.org/E42F47F0-E9D1-492D-9D88-9E02163BA2D5

Figs 5–7 , 8–16 , 17–32 , 33–42

##### Type material examined.

***Male Holotype.*** Colombia: Caquetá, Morelia, Vda. Caldas, Fca. El Porvenir; 01°29'57"N, 75°44'03"W, 272 m, 05-Dec.-2021, A. Gamboa// Captura con jama entomológica en dosel (hoja) de [Collected with entomological net in canopy (leaf) of] *Theobromacacao* (Malvaceae)-cacao//LEUA-42920//[red printed label:] HOLOTYPE (LEUA).

##### Diagnosis.

*Neotropiconyttusarmandoi* sp. nov. can be distinguished from the congeneric species by the orange pronotum with darker orange symmetrical spots on the anterior lobe (Fig. 10).

##### Description.

**Male. Measurements (mm)**: Body length: from frons to tip of hemelytra 11.52; to tip of abdomen 8.42. ***Head***: length 1.74; anteocular portion (lateral view) 0.39; postocular portion (lateral view) 0.49; head width across eyes 1.56; interocular distance 0.76; eye width 0.41; eye length 0.71; ocellar tubercle width 0.26. Antenna: scape length 3.52; pedicel length 0.86. Labium (lateral view): visible segment II length 0.98; labial segment length III 0.68; labial segment length IV 0.40. ***Thorax***: pronotum length (at midline) 1.95; pronotum maximum width 2.23; scutellum length (at midline) 0.59. Hemelytron: total length 8.28; membrane 4.79. Legs (lateral view): foreleg: coxa 0.64; trochanter 0.12; femur 3.31; tibia 3.41; tarsus 0.42; middle leg: coxa 0.26; trochanter 0.21; femur 2.76; tibia 3.32; tarsus 0.45; hind leg: coxa 0.27; trochanter 0.17; femur 3.74; tibia 4.73; tarsus 0.51. ***Abdomen***: total length (ventral view, at midline, from anterior margin of sternite II to posterior border of genitalia): 3.88; maximum width 1.49.

**Figures 5–7.**
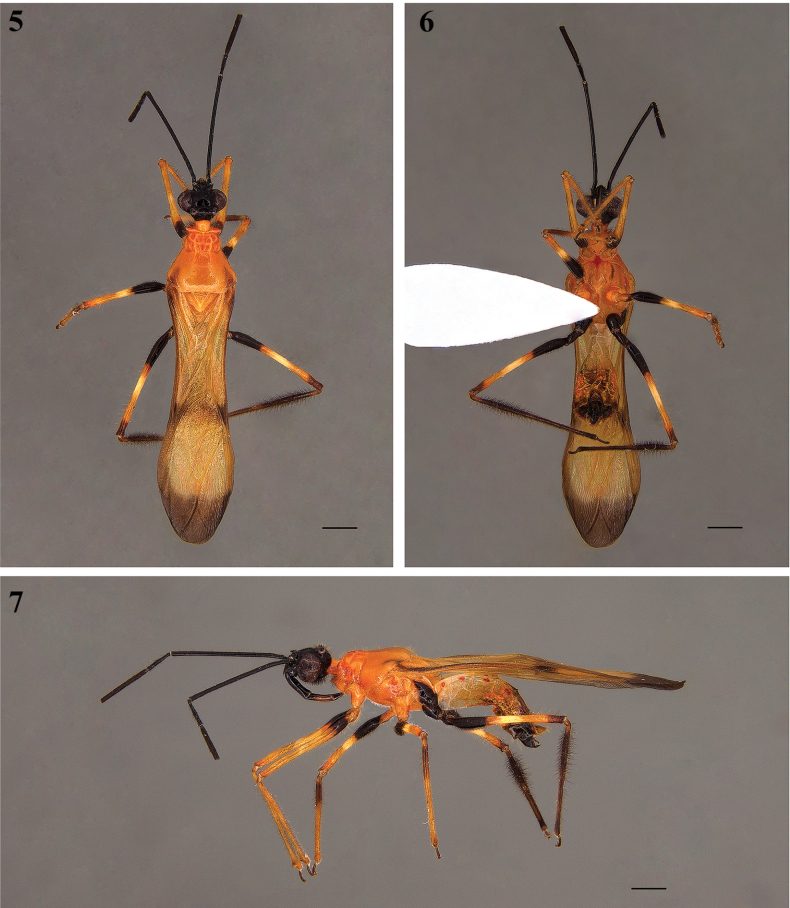
*Neotropiconyttusarmandoi* sp. nov., holotype, male habitus **5** dorsal view **6** ventral view **7** lateral view. Scale bars: 1.0 mm.

**Figures 8–16.**
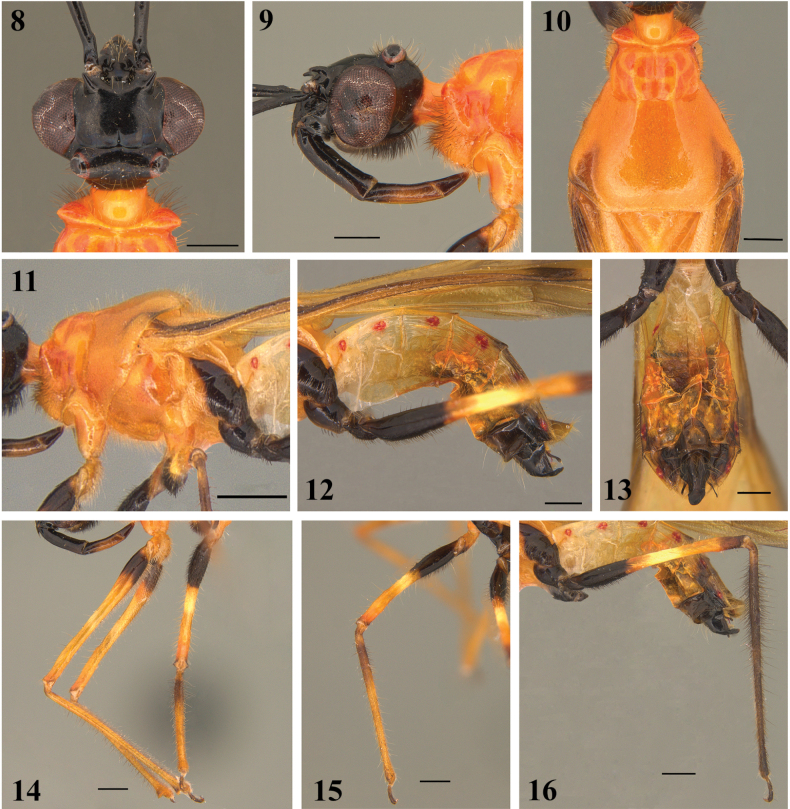
*Neotropiconyttusarmandoi* sp. nov., holotype, male **8, 9** head **8** dorsal view **9** lateral view **10, 11** thorax **10** dorsal view **11** lateral view **12, 13** abdomen **12** lateral view **13** ventral view **14** foreleg and a middle leg **15** middle leg **16** hind leg. Scale bars: 1.0 mm (**11**); 0.5 mm (**8–10**, **12–16**).

**Coloration: *Head***: mostly black; neck orange; ocellar tubercle paler around each ocellus; eyes dark brown (Figs 5–9); antenna [distal portion absent]: scape, pedicel, and basal portion of first flagellomeres black (Figs 5–7); labium: visible segments: II black, III mostly pale brownish, blackish basally and slightly darker at apex, IV dark brown (Fig. 9). ***Thorax***: mostly orange, somewhat paler at pleural and sternal areas; pronotum, collar, anterior lobe of pronotum with faint reddish tinge; darker orange symmetrical spots (dorsal part) and small reddish dots (lateral and ventral) on anterior lobe; posterior lobe of pronotum orange with inner portions of humeral areas pale brown; scutellum pale orange; mesepisternum orange with small lateral reddish dots and long reddish spot anteroventrally; metepisternum orange anteriorly and black posteriorly, above the hind coxa (Figs 10, 11). Hemelytron: generally orange, somewhat translucent, with anterobasal angle, costal margin and a narrow transversal stripe, fainter at median portion and approximately apical ~ 1/4 of membrane pale blackish (Figs 5–7). Legs: fore and middle coxae orange and trochanters orange basally and darker distally; hind coxae and trochanters blackish. Femora generally orange; from fore to hind femora basal portion progressively more extensively dark brown to blackish; approximately at middle, a submedian ill-defined narrow pale annulus; apices somewhat darkened, more extensively on hind femora. Fore and middle tibiae orange, the former with small basal and distal dark markings, latter with extreme base and a large subbasal portion dark; hind tibiae pale blackish, apex paler; tarsi dark (Figs 7, 14–16). ***Abdomen***: pale orange (sternites II‒IV), darker orange (sternites V and VI), and blackish (sternite VII), spiracles with the area surrounding them reddish, pygophore blackish (Figs 12, 13).

**Structure: *Head***: 1.10× as long as wide, labrum triangular 1.38× as long as wide, postclypeal suture deep, frons 0.51× as wide as head width (anterior view), antennal sclerite slightly raised, postantennal spine curved forward, small, apex acute. Eyes large, protruding, glabrous, rounded, and ellipsoid in dorsal and lateral views, respectively, reaching closer and beyond dorsal and ventral margins of the head; postocular region short; ocelli and area between them elevated, the former much closer to eyes than to each other; ellipsoid on tubercles, tubercles anteriorly bounded by postocular suture, which is thin and shallow, curved laterally; just anterior to it, on midline, a small oval fossa; mandibular plate triangular, maxillary plate slightly bulged, postgena reduced, flat posteriorly, gula slightly swollen (Figs 8, 9); antenna: scape cylindrical, long, somewhat curved; 2.02× as long as head length, base flattened; pedicel 0.24× as long as scape, short, thinner basally; basal portion of basiflagellomeres [the portion still present in the holotype] cylindrical, straight, slightly thicker than scape; remaining portions of basiflagellomere and both distiflagellomeres absent. Labium: [visible] segment II cylindrical, basally curved, 0.47× as long as labium; III slightly curved and reduced, 0.33× as long as labium; IV conical, 0.19× as long as labium. ***Thorax***: collar, 0.02× as long as pronotum at midline, lateral areas longer, subtriangular, in dorsal view; pyramid-shaped in lateral view; anterior lobe of pronotum 0.30× as long as pronotum total length, with mesial longitudinal suture deep, absent on hind lobe; lateral portions of anterior lobe slightly swollen; transverse sulcus of pronotum not deep, interrupted submedially by a pair of shallow carinas straight and curved between and laterally to the latter, respectively. Hind lobe with disc smooth, lateral longitudinal sulci well marked at posterior 1/2 to 2/3; humeral angle moderately elevated, rounded at lateral margin. Scutellum with margins elevated, apex thin, acutely pointed. Pleural suture long; epimeron concave; mesoepisternum swollen; metaepisternum rhomboid (lateral view); metepimeron medial lobe narrowing posteriorly (Figs 10, 11). Hemelytron: 2.13× as long as total abdomen length; membrane ellipsoid, reaching 0.57× hemelytron length total, 2.21× as long as width. Legs: coxa truncated cone-shaped; trochanters, slightly curved; femora enlarged at basal portion and slightly thickened distally, apices with short anterior and posterior projections; tibia generally cylindrical; fore tibia slightly curved basally, enlarged apically where there is a small spur and a mesial comb; middle tibiae straight, slightly thickened at apex; hind tibiae somewhat enlarged at subbasal 1/3; tarsi thickening distally towards the apex (Figs 14–16). ***Abdomen***: 1.98× as long as pronotum total length. Dorsal aspect, segments II‒VII, each from 0.12× to 0.17× as long as abdomen total length, and pygophore 0.09× as long as abdomen total length. Posterior margin of segment VIII exposed ventrally, wider laterally, and shorter at median portion (Figs 12, 13).

**Figures 17–32.**
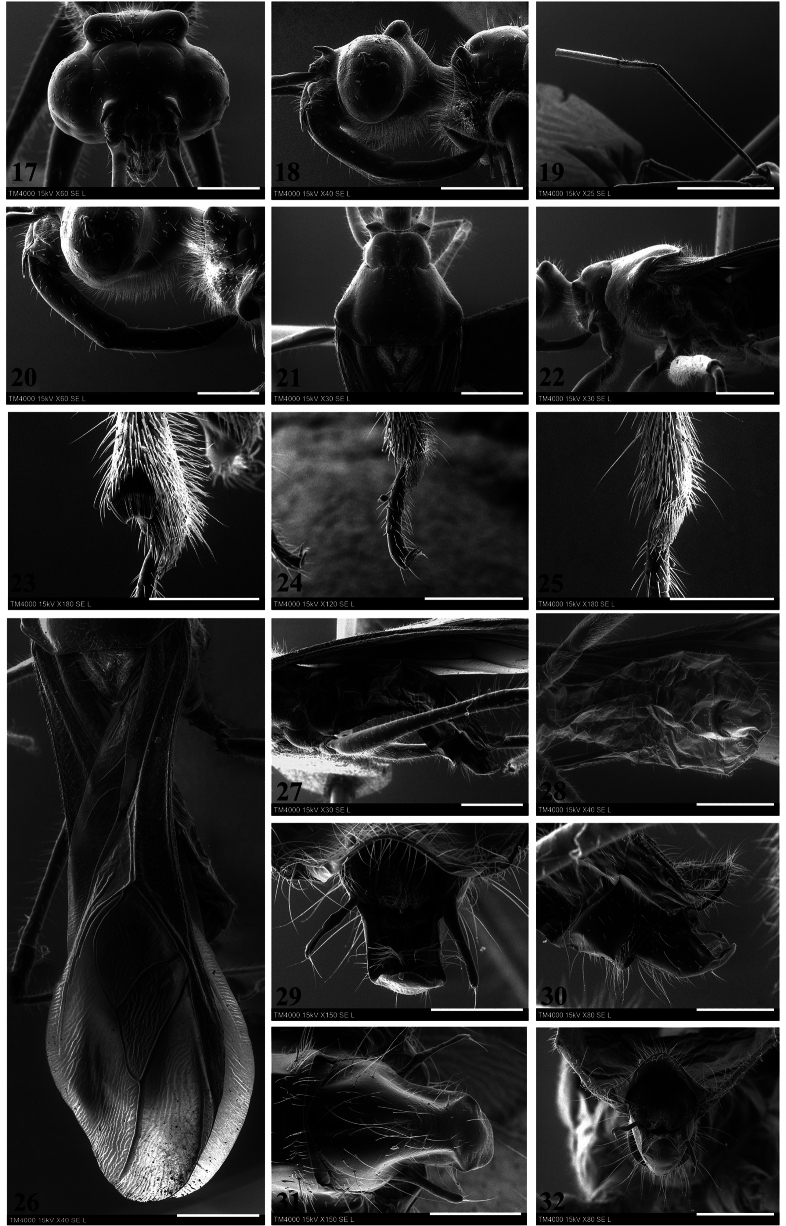
*Neotropiconyttusarmandoi* sp. nov., holotype, male **17** dorso-anterior view head **18** lateral view head **19** antenna **20** labium and ventral portion of head and, lateral view **21, 22** thorax **21** dorsal view, **22** lateral view **23–25** inner surface of apical portion of tibiae **23** fore tibia **24** middle tibia **25** hind tibia **26** posterior margin of pronotum scutellum and hemelytra **27** lateral view abdomen **28** ventral view abdomen **29–32** pygophore **29** dorsal view **30** lateral view, including the VII and posterior margin of VIII abdominal segments **31** ventral view **32** posterior view, including the dorsal portion of VII abdominal segment. Scale bars: 300 µm (**23, 25, 29, 31**); 400 µm (**24**); 500 µm (**17, 20, 30, 32**); 1.0 mm (**18, 21, 22, 26–28**); 2.0 mm (**19**).

**Vestiture: *Head***: labrum glabrous; clypeus, gena, mandibular plate, maxillary plate, and buccula with suberect and slightly pale curved setae, on clypeus distributed on the lower 2/3; frons mostly glabrous with three setae between postantennal spines; vertex mostly glabrous with very few pale setae near margins surrounding eyes; ocellar tubercles with suberect, slightly curved pale and some darkened setae; postocular region with few erect and curved pale and some darkened setae on dorsal and lateral areas; neck glabrous; gula with thick and curved and denser blackish setae on median portion, forming a pubescence (Figs 17, 18). Antenna: scape with few slightly curved, suberect setae, shorter than scape width; pedicel, except the glabrous base, covered by numerous erect and suberect, short and long, straight and curved dark setae, length subequal to pedicel diameter, one seta longer and thinner than others on apex (Fig. 19). Labium: segment II (first visible) with few curved pale setae, on anterior portion, although variable in their length, all shorter than segment diameter; III and IV with very few curved, short, pale setae (Fig. 20). ***Thorax***: anterior margin of prothorax, including collar, propleura and sternal portion, densely setose, forming a pubescence of long, thin, somewhat darkened setae; midline of collar with long sparser pale setae; remaining portions of pronotum generally covered by scattered thin, moderately long, curved or straight pale setae, somewhat more numerous on posterior margin; anterior portions of propleura with numerous long, thin, darkened setae; proepimeron setose; mesoepisternum with setae, denser and longer mesially; mesepimeron covered with very dense short setae; metepisternum with denser setae ventrally (Figs 21, 22). Hemelytron: clavus with short, pale, decumbent, curved setae, which are more numerous on basal portion; corium with similar setae more numerous over veins; membrane glabrous (Fig. 26). Legs: coxae: setae suberect, curved, at least with three different sizes, maximum length 1/2 of coxa diameter in lateral view, on foreleg very numerous on posterior surface, on middle and hind legs very numerous on anterior and posterior surface; trochanter with numerous setae on anterior, mesial, and posterior surfaces, maximum length subequal to coxa diameter in lateral view. Femora, with setae of at least three lengths, some longer than femur width; on basal portion, they are more numerous, forming a pubescence of dense, erect, brush-like setae ventrally; laterally few setae. Tibia covered by many suberect, subdecumbent, and decumbent setae, except basally; setae longer and more abundant proximally, fore, middle, and hind tibiae with long setae densely packed on the tibia apex in medial surface (Figs 23–25). Setae on tarsi suberect, several of which longer than twice tarsus diameter. ***Abdomen***: lateral margin of tergites II‒VI with very few, short, curved, and suberect setae; tergite VII with numerous setae of different sizes; sternites II‒VII with setae on ventral side of different sizes, curved, and suberect, some setae slightly shorter than parameres length, sternite VIII glabrous (Figs 27–32).

**Figures 33–42.**
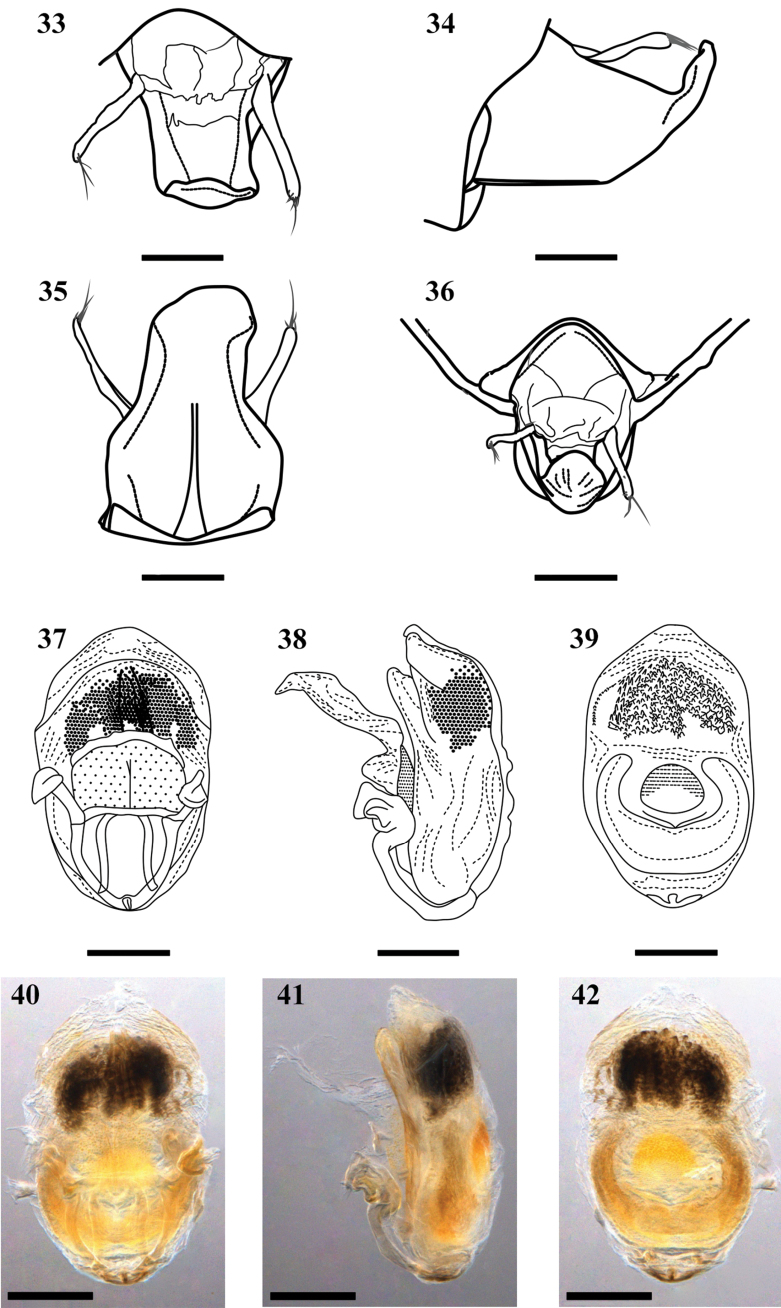
*Neotropiconyttusarmandoi* sp. nov., holotype, male genitalia **33–36** pygophore and parameres, general outline **33** dorsal view **34** lateral view **35** ventral view **36** posterior view **37–39** aedeagus **37** dorsal view **38** lateral view **39** ventral view **40–42** aedeagus with endosoma completely inflated **40** dorsal view **41** lateral view **42** ventral view. Scale bars: 0.3 mm (**36**); 0.2 mm (**33–35, 37–42**).

**Genitalia**: Pygophore: mostly black, in dorsal view: 1.12× as long as maximum (anterior) width, concave, slightly narrower in middle and posterior portions; in ventral view wider anteriorly, anterior surface 0.67× as wide as total length, ventrally swollen, with anterior mesial folds extending posteriorly for 0.43× the total length of pygophore; in lateral view, scalene-triangle-shaped, subtrapezoidal, posterior margin upwards. Vestiture: setae yellowish orange, curved, subdecumbent, and with different lengths, several slightly longer than 0.75× pygophore length (lateral view), more numerous on ventral surface (Figs 29–36). Parameres: dark brown and black, symmetrical, elongated; right paramere broad basally, cylindrical both proximal and distally, rounded apically, apex with erect and slightly curved setae (~ 8) of different lengths, distal 1/2 with a dorsal seta decumbent and short; left paramere slightly warped from drying (Figs 29–36). Phallus: flat dorsoventrally. Articulatory apparatus (dorsal view): basal plate, in dorsal and lateral views, with subrounded arms and with 1/2 to 1/3 of the length in comparison of that of the phallus, inflected distally, basal plate bridge 0.58× as long as right arm of the articulatory apparatus (Figs 37–42). Dorsal phallothecal plate: elongated, weakly sclerotized, subrectangular in dorsal view, elongated posteriorly, slightly curved anteriorly, proximal part with punctures, distal part smooth, laminate and translucent, and with wrinkled margins; struts with subparallel arms slightly curved, joined distally (Figs 37–42). Endosoma: endosoma wall translucent, faintly rugous, slightly wrinkled apically. Three processes of endosoma: a large pale U-shaped to subrounded basal process formed by diffuse thickening; a median subspherical process lying between the lateral arms of the U-shaped basal process and formed by a grouping of small thickenings and a subdistal large darkened endosoma process formed by numerous small acute processes (Figs 37–42).

##### Etymology.

*Neotropiconyttusarmandoi* sp. nov. is named in memory of Armando Gamboa Torres (1955–2007), father of the first and third authors herein. Armando was a primary and secondary school teacher who, every day after his long working hours, devoted his time to agriculture. As time went by, he gathered an important set of empirical knowledge of crops in traditional agroecosystems of the Amazon region, such as banana (*Musa* spp. - Musaceae), cassava (*Manihotesculenta* Crantz - Euphorbiaceae), sugarcane (*Saccharumofficinarum* L. - Poaceae), and corn (*Zeamays* L. - Poaceae). Thereby, Armando ingrained his interest in agriculture throughout his life in all “his” kids.

##### Distribution.

Colombia (Caquetá).

##### Type locality.

Colombia, Caquetá, Morelia, Vda. Caldas, Fca. El Porvenir, 01°29'57"N, 75°44'03"W 272 m.

#### ﻿ Neotropiconyttus dama

Taxon classificationAnimaliaHemipteraReduviidae

(Burmeister, 1838)

E0341C0B-2AE6-5D52-8390-3DC3E378484D

Figs 43–51

Myocoris dama Burmeister, 1838: 105 [description]; Walker 1873: 130 [catalog].
Amaurosphodrus dama ; Stål 1872: 82 [catalog]; Lethierry and Severin 1896: 178 [catalog].
Neotropiconyttus dama ; Wygodzinsky 1949: 42 [catalog]; Maldonado 1990: 241 [catalog]; Maldonado and Lozada 1992: 165 [comparison with other species based on color characteristics].

##### Distribution.

Brazil.

##### Type material examined.

*Myocorisdama* Burmeister, 1838. Brazil: three female syntypes: [printed label] 2780 // [handwritten green label] *Dama* / *N.* // [handwritten green label] Parà [Pará] Sieber // [printed red label] Typus; [handwritten] 2780 // [handwritten] ^x^*Neotropiconyttus* / *dama* (Burm.) / Paratypus ♀ // [handwritten green label] Para / Sieber // [printed red label] Paratypus; [handwritten] 2780 // [handwritten] ^x^*Neotropiconyttus* / *dama* (Burm.) / Paratypus ♀ // [handwritten green label] Para / Sieber // [printed red label] Paratypus (MFNB).

**Figures 43–51.**
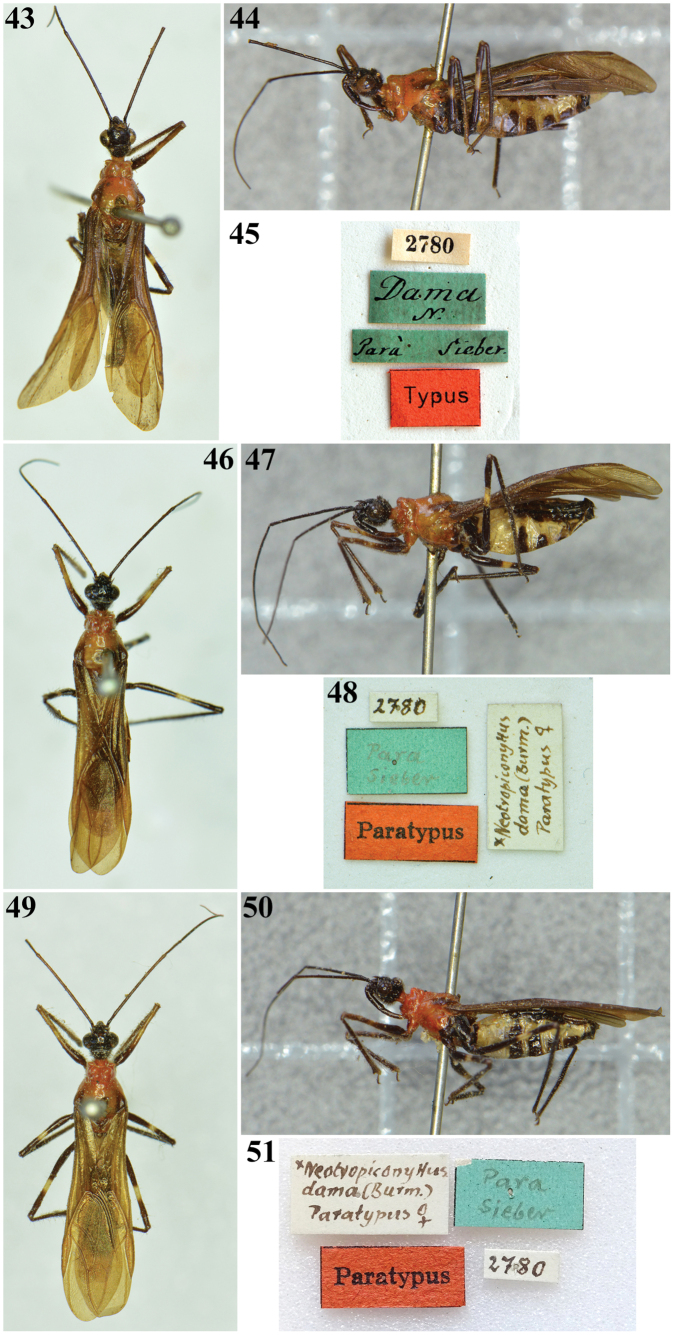
*Neotropiconyttusdama* (Burmeister, 1838), syntypes, females **43–45** specimen labeled as “Typus” **43** dorsal view **44** lateral view **45** labels **46–51** specimens labeled as “Paratypus” **46** dorsal view **47** lateral view **48** labels **49** dorsal view **50** lateral view **51** labels.

*Neotropiconyttusdama* was described based on specimens from the State of Pará, Brazil (Burmeister 1838). In the MFNB, there are three female type specimens of *N.dama*. One specimen is labeled as “Typus” (Figs 43–45); the others are labeled as “Paratypus” (Figs 46–51). All of them are considered as syntypes in this work, following Art. 73.2 of the ICZN (1999). Notably, all the syntypes have a green label stating, besides the name of the Brazilian state of Pará, the name of “Sieber.” Friedrich Wilhelm Sieber was a servant and preparator of Johann Centurius Count von Hoffmannsegg, who obtained permission from the King of Portugal to visit Brazil to collect insects. Leaving Lisbon in 1801, Sieber went to the Province of Pará, where he stayed for 12 years, collecting not only in the vicinity of Belém but also in different areas of the provinces of Pará and Rio Negro (currently, state of Amazonas) (Papavero 1971). Therefore, the precise type locality of the species in this large region remains uncertain.

##### Morphological remarks.

General length 12‒12.5 mm. General color blackish; labium with distal 1/2 of second and third visible labial segments variably paler; neck, prothorax, and mesothorax mostly reddish; humeral angles, posterior margins of mesopleura and mesosternum, and most of metapleura dark to blackish. Legs: except for fore coxae and a portion of fore trochanters, which are reddish, the remaining portions of the legs mostly dark to blackish; dorsal portion of distal ~ 2/3 of fore femora paler, and ill-defined pale yellowish annuli; middle and hind femora with submedian distal pale whitish to yellowish annuli; hemelytra darkened. Abdomen: sternites mostly yellowish with the following portions or markings blackish: narrowly basally on sternite II and adjacent connexival portion; on segments IV‒VII: connexivum and shortly adjacent portion, median markings of variable extension on respective sternites, and genitalia.

### ﻿Key to species of *Neotropiconyttus*

**Table:** 

1	Anterior lobe of pronotum orange with darker orange symmetrical spots (Fig. 5)	***N.armandoi* sp. nov.**
–	Anterior lobe of pronotum black or reddish (Figs 1, 43, 44, 46, 47, 49, 50)	**2**
2	Posterior lobe of pronotum black and ivory white	***N.heminigra***
–	Posterior lobe of pronotum entirely black or reddish with humeral angles dark to blackish (Figs 1, 43, 44, 46, 47, 49, 50)	**3**
3	Pro- and mesothorax mostly reddish or orange, abdomen mostly yellow to pale whitish, segments IV‒VII with connexivum, large median dark markings on sternites, and genitalia blackish (Figs 43, 44, 46, 47, 49, 50)	***N.dama***
–	Thorax entirely black, abdomen entirely reddish at anterior ~ 1/2 and posteriorly blackish (Figs 1–3)	***N.alboannulatus***

## ﻿Discussion

### ﻿Identification

*Neotropiconyttus*, *Myocoris*, *Xystonyttus*, *Graptocleptes*, *Hiranetis*, *Parahiranetis*, and *Quasigraptocleptes* can be recognized by following the wasp-mimic Harpactorini key of Gil-Santana and Oliveira (2023). The remarkable similarity in shape, coloration, size, and setosity among the specimens representing the genera mentioned above has led to errors in the identification and cataloging at several entomological collections (personal observation of the first two authors), and unexpected synonymies, such as that recorded by Gil-Santana et al. (2013).

The three previously recognized species of *Neotropiconyttus* differ in coloration on the pronotum, mesosternum, mesopleuron, fore trochanter, hemelytron, and abdomen (Maldonado and Lozada 1992). A taxonomic revision of the genus based on more anatomical characters would be necessary to delimit the species better. We suggest exploring a new set of characters that could be employed to describe or redescribe the species, including measurements, detailed coloration, and genitalia traits.

**Figures 52–56.**
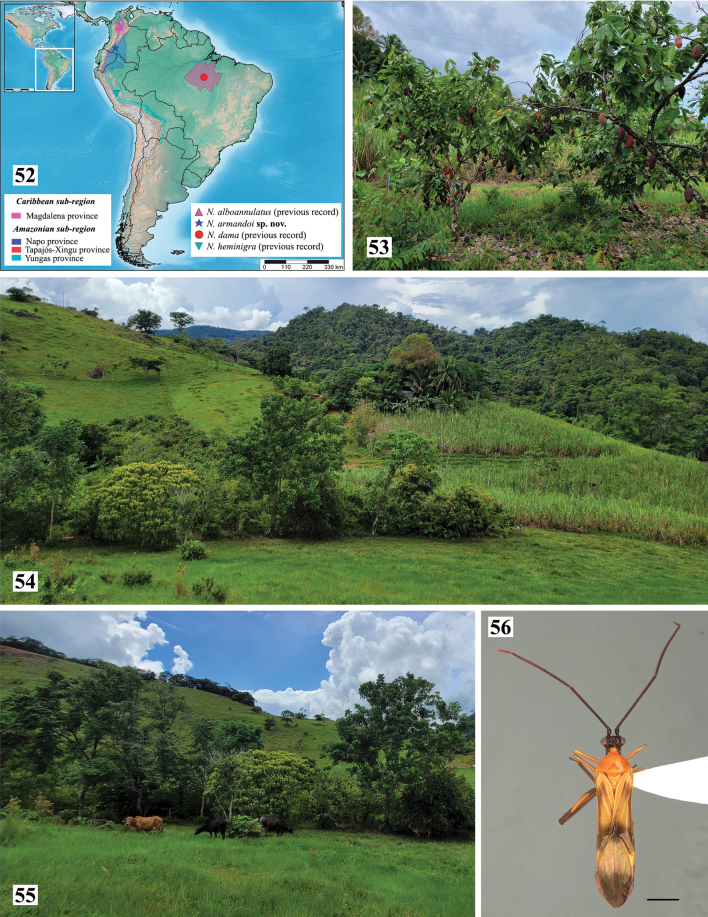
*Neotropiconyttusarmandoi* sp. nov., habitat **52** geographical distribution of *Neotropiconyttus* species **53**cacao agroecosystem (type locality) **54** adjacent agroecosystems of *Saccharumofficinarum* (Poaceae) and *Brachiariadecumbens* (Poaceae) **55** live fence with native trees and shrubs **56***Monaloniondissimulatum* Distant, 1883, male habitus. Scale bar: 2.0 mm.

All species of *Neotropiconyttus*, including *N.armandoi* sp. nov., were described based on specimens from a single locality and few additional specimens were observed after the original descriptions (e.g., *N.alboannulatum* by Champion 1899). This circumstance adds some taxonomic issues since population variation could not be recorded. The scarce data regarding the distribution of *Neotropiconyttus* species (Fig. 52) is due to sampling bias. More collecting effort is necessary to better understand the distribution and dispersion of the species.

### ﻿Habitat

*Neotropiconyttusarmandoi* sp. nov. is only known from the type locality in Morelia, Caquetá, Colombia, in the transition zone between Cordillera Oriental (eastern mountain range) and the Amazonian basin, corresponding to the Napo province of Colombia. The previous three recognized *Neotropiconyttus* species are recorded from localities in Brazil (Pará), Perú (Iscozacin), and Colombia (Remedios, Antioquia). Morelia, Pará, and Iscozacin correspond to localities in the provinces of Napo, Tapajós-Xingú, and Yungas, respectively, in the Amazonian subregion; and Remedios corresponds to the Magdalena province, in the Caribbean subregion (Fig. 52) (Morrone 2014).

The type locality of *Neotropiconyttusarmandoi* sp. nov. is part of one of the ecoregions with the highest deforestation areas, in which the forest is felled and burned to establish introduced pastures for livestock. In this ecoregion, there is a prevailing need to recognize insect diversity, understand the impacts of anthropic activities on this biological group, and design and implement environmental conservation strategies.

The only known individual of the species was associated with an agroforestry system that includes trees and bushes, such as *Theobromacacao* L. (Malvaceae), *Eugeniastipitata* McVaugh (Myrtaceae), and *Musaparadisiaca* L. (Musaceae), bordered by crops of *Saccharumofficinarum* L. (Poaceae) and *Brachiariadecumbens* Cv. Basilisk (Poaceae) (Figs 53, 54). This ecosystem has ecological connectivity through living fences in riparian vegetation (native trees and shrubs) of a lotic ecosystem (Quebrada La Sardina) (Fig. 55).

The *Neotropiconyttusarmandoi* sp. nov. individual was collected inside an agroforestry system, standing on a *T.cacao* tree leaf near the fruits of this crop, in which individuals of the true bugs of the genus *Monalonion* feed, with a predominance of the species *Monaloniondissimulatum* (Fig. 56). The astonishing similarities between the two species regarding size, shape, and coloration characters prompted the inference of mimicry between *Neotropiconyttusarmandoi* sp. nov. and *M.dissimulatum*.

## Supplementary Material

XML Treatment for Neotropiconyttus

XML Treatment for Neotropiconyttus alboannulatus

XML Treatment for Neotropiconyttus armandoi

XML Treatment for Neotropiconyttus dama

## ﻿Additional information

### Author ORCIDs

Jean Gamboa https://orcid.org/0000-0002-8783-0175

Hélcio R. Gil-Santana https://orcid.org/0000-0002-0544-348X

Armando Gamboa-Tabares https://orcid.org/0000-0001-5777-8495

Eidy Martínez-Viuche https://orcid.org/0009-0001-6558-6482

Francisco Serna https://orcid.org/0000-0002-6143-9821

### Data availability

All of the data that support the findings of this study are available in the main text.
